# Factors Associated with Antibiotic Initiation After DaBT-Hib-IPV Vaccination in NICU-Hospitalized Preterm Infants: The Role of C-Reactive Protein

**DOI:** 10.3390/jcm15155934

**Published:** 2026-07-29

**Authors:** Hüseyin Şimşek, Mustafa Akçalı, Mehtap Durukan Tosun, Berfin Özmen, Suna Özdem

**Affiliations:** 1 Neonatology Department, Mersin City Training and Research Hospital, 33240 Mersin, Türkiye; akcali_mustafa@hotmail.com (M.A.); mehtap.durukan@gmail.com (M.D.T.); 2 Department of Pediatric Infectious Diseases, Mersin City Training and Research Hospital, 33240 Mersin, Türkiye; dr.b.ozmen@hotmail.com (B.Ö.); sunaozdem@hotmail.com (S.Ö.)

**Keywords:** antibiotic stewardship, C-reactive protein, infant, sepsis, vaccine

## Abstract

**Objective**: The purpose of this study was to evaluate the factors, particularly post-vaccination C-reactive protein (CRP) and the neutrophil-to-lymphocyte ratio (NLR), associated with the clinical decision to initiate antibiotic treatment following the 2-month diphtheria–acellular pertussis–tetanus–Haemophilus influenzae Type B–inactivated poliovirus (DaBT-Hib-IPV) vaccine in hospitalized infants. **Methods**: A retrospective review was conducted on the medical records of 46 infants. The study included infants hospitalized between 2022 and 2024 at Mersin City Training and Research Hospital who received the 2-month vaccine and subsequently underwent post-vaccination laboratory testing. Infants were categorized based on whether they received antibiotic treatment. **Results**: Antibiotics were administered to 20 infants, and 26 were observed without any treatment. Despite having a significantly higher gestational age (*p* = 0.027), the hospital stays of the antibiotic group were significantly longer (median 99 vs. 78 days, *p* < 0.001). C-reactive protein (CRP) and the neutrophil-to-lymphocyte ratio (NLR) were elevated in the antibiotic group (CRP = 2.82 vs. 1.63 mg/dL, *p* < 0.001; NLR= 0.90 vs. 0.72, *p* = 0.041). Culture positivity was low in antibiotic recipients (3/20, 15%). CRP was the only independent predictor of antibiotic administration when adjusted for gestational age and weight at vaccination (Exp(B) = 3.997, *p* = 0.003). **Conclusions**: In this cohort of NICU-hospitalized, predominantly preterm infants, a population that does not represent the general population of vaccinated 2-month-old infants, post-vaccination elevations in CRP and NLRs were associated with the clinical decision to initiate antibiotic treatment; however, in the absence of a validated infection reference standard, these markers cannot be assumed to reliably differentiate true infection from vaccine-induced inflammation. To prevent unnecessary antibiotic use and optimize patient management, these markers must be evaluated in conjunction with clinical findings, culture results, and supplementary biomarkers.

## 1. Introduction

Neonatal sepsis is characterized by clinical signs of systemic infection, elevated infection markers in laboratory tests, and sometimes the isolation of the causative agent in blood cultures [1,2]. Given the absence of pathognomonic clinical signs or diagnostic biomarkers [3], the diagnosis of neonatal sepsis requires a low threshold of suspicion and a high level of awareness [4]. While useful for diagnosis, infection markers like white blood cell (WBC) count, C-reactive protein (CRP), and procalcitonin may be influenced by infants’ physiology, immaturity of the immune response, and outside factors—which include routine vaccinations [5]. Indeed, vaccines are key immunologic stimuli in early infancy and may complicate the assessment of sepsis risk in suspected cases. For instance, some infants receiving the diphtheria–acellular pertussis–tetanus–Haemophilus influenzae Type B–inactivated poliovirus (DaBT-Hib-IPV) vaccine at 2 months of age may develop fever, irritability, feeding difficulties, or transient laboratory abnormalities. Since these changes could easily be mistaken for development of an overt infection, careful clinical assessment is necessary [6]. It is important to emphasize that DaBT-Hib-IPV is a component of the Turkish National Immunization Program and is essential for protecting vulnerable hospitalized infants from vaccine-preventable disease. The present study does not imply that vaccination causes infection; rather, it addresses the diagnostic challenge that transient, expected post-vaccination immune activation poses when clinicians must distinguish it from a coincidentally evolving true infection in the same time window. Not only is this critical for suspecting sepsis but also for the decision to initiate antibiotic therapy, especially because avoiding unnecessary antibiotic use in non-septic conditions is essential to prevent antimicrobial resistance and treatment-related adverse effects in this vulnerable age group [7].

Infants may experience transient post-vaccination clinical events after routine 2-month immunization; however, these events do not necessarily indicate serious infection or justify deviation from routine vaccination schedules [8]. During the assessment of suspect cases in this period, inflammatory markers must be monitored/assessed with respect to their defining properties. For instance, CRP is one of the most important markers in this sense and becomes detectable 10–12 h after infection onset, peaking at 36–48 h, and can be used to monitor treatment response [9]. Well-established causes of elevation in the neonatal period and infancy include maternal fever, premature rupture of membranes, difficult delivery, meconium aspiration, perinatal asphyxia, pneumothorax, intracranial hemorrhage, steroid use, and vaccination [10]. Since singular parameters are insufficient in the decision-making process, particularly for infants, it may be relevant to assess whether composite measures can address these deficiencies.

As a simple ratio derived from the routine complete blood count, NLR reflects the balance between innate immune activation, marked by neutrophilia, and the relative lymphopenia that accompanies systemic inflammatory and infectious states, and it has the practical advantage of requiring no additional testing beyond a standard hemogram [11]. Because CBC-derived indices are inexpensive and rapidly available, they have been proposed as accessible alternatives to costlier inflammatory biomarkers in resource-conscious settings [12], and their component parameters carry distinct pathophysiological meaning that supports their use as composite inflammatory indices [13]. Comparative studies evaluating the NLR against other CBC-derived indices such as the SII have shown that neither consistently outperforms the other across all clinical contexts, indicating that their discriminative value is likely population- and disease-specific [14]. In a Turkish cohort, CBC-derived ratios including the NLR were able to distinguish infected from non-infected patients, although the direction and magnitude of change varied with the underlying condition and population studied [15], underscoring the need for validation of these markers in population- and context-specific settings such as the vaccinated, hospitalized preterm infant described here. Whether these adult-derived observations extend to the neonatal population, and specifically to the post-vaccination period, has not been well characterized, which is a central motivation for the present study. The advantage of composite measures such as the SII is that they can be easily calculated from routine complete blood count parameters and may aid in the differentiation of infection from immune response in infants with post-vaccination clinical deterioration.

Post-vaccination cardiorespiratory events, including apnea, bradycardia, and oxygen desaturation, are well described in preterm infants and are more common at lower gestational age and birth weight, suggesting that they represent a nonspecific physiological response to medical intervention rather than a vaccine-specific reaction [16,17,18]. In one of the few available reports specifically addressing laboratory correlates, transient CRP elevations were observed in premature infants following routine immunization, particularly after simultaneous administration of multiple vaccines [18]. However, it remains poorly characterized whether CRP and the NLR generally follow a similarly self-limited pattern in hospitalized preterm infants receiving combination vaccines, or whether such elevations should instead prompt investigation for true infection. This gap is clinically important, as it directly affects antibiotic-initiation decisions in a population already vulnerable to nosocomial infection and antimicrobial resistance.

This study aimed to identify clinical and laboratory factors, with particular focus on post-vaccination CRP and the NLR, associated with the decision to initiate antibiotic treatment in hospitalized infants following the DaBT-Hib-IPV vaccine. The study was designed to characterize post-vaccination trends in inflammation markers and provide guiding evidence that could inform the decisions for antibiotic use in this clinical setting.

## 2. Materials and Methods

### 2.1. Study Design and Ethics

This was a retrospective cohort study including infants hospitalized in the neonatal intensive care unit (NICU) of a tertiary hospital between 1 January 2022, and 31 December 2024. All infants underwent post-vaccination assessment of infection markers following the routine 2-month immunization, which included the combined diphtheria, tetanus, acellular pertussis, inactivated poliovirus, and *Haemophilus influenzae* type b vaccine (Pentaxim^®^, Sanofi Pasteur, Lyon, France). Clinical characteristics, laboratory results, and outcomes were extracted retrospectively from electronic medical records, infection consultation notes, nursing documentation, and the hospital information system.

The Toros University Scientific Research and Publication Ethics Committee approved this study (Date: 23 June 2025, Decision no: 06/109). The requirement for informed consent was waived due to the retrospective nature of the study. All personal identifiers were removed prior to data analysis to ensure patient confidentiality. The study was performed in accordance with the principles of the Declaration of Helsinki.

### 2.2. Participant Selection and Classification

Eligible infants were those who received the 2-month vaccination while in the NICU, remained hospitalized for at least 96 h post-vaccination, and had blood tests performed between 48 and 72 h after vaccination. Exclusion criteria were: prior antibiotic use before vaccination, absence of post-vaccination laboratory tests, discharge within 24–48 h after vaccination, use of immunosuppressive medications, drugs affecting complete blood count parameters, receipt of blood products, recent injections, and incomplete medical records. Participants were classified into two groups: (1) infants who received antibiotics after vaccination and (2) infants who did not receive antibiotics.

Infection status was classified according to the 2018 Turkish Neonatal Society Guidelines for Diagnosis and Treatment of Neonatal Infections [19]. Proven sepsis was defined as clinical and laboratory findings consistent with sepsis together with identification of a causative organism; clinical sepsis was defined as clinical and laboratory findings consistent with sepsis without an identified organism. Coagulase-negative staphylococci (*Staphylococcus epidermidis*) isolated from a single culture were considered probable contaminants when a repeat culture was negative, but antibiotic treatment was not discontinued early per unit protocol. Post-vaccination laboratory testing was prompted by clinical findings occurring within 48–72 h after vaccination, including tachycardia, fever, irritability, apnea, increased oxygen requirement, vomiting, cutaneous changes, or decreased activity. Testing was not guided by a fixed institutional protocol but was based on individual clinical assessment.

All infants in this cohort were born preterm and remained hospitalized in the NICU beyond 2 months of chronological age. This continued hospitalization reflected ongoing needs related to prematurity, including growth monitoring, resolution of respiratory distress, follow up of intraventricular hemorrhage, and establishment of maternal infant bonding prior to discharge, rather than acute illness at the time of vaccination.

### 2.3. Data Collection

Age, sex, birth weight, length of hospital stay, infection development, and laboratory values (WBC, CRP, neutrophil, platelet, lymphocyte, NLR, and SII) were assessed. We also collected data concerning infection detection and/or management approaches, including culture results and treatment regimens administered to those who were deemed to require antibiotics. Per standard unit protocol, blood cultures were routinely obtained prior to antibiotic initiation.

Laboratory data to assess inflammatory activity was obtained from blood samples collected as part of patient management and quantified using routine devices calibrated according to international standards. The NLR was calculated by dividing neutrophil count by lymphocyte count. The SII was calculated using platelet (reference range: 150–400 × 10^3^/µL), neutrophil (reference range: 1.8–6.98 × 10^3^/µL), and lymphocyte (reference range: 1.26–3.35 × 10^3^/µL) values, with the following formula: SII = (Platelet count × Neutrophil count)/Lymphocyte count.

Complete blood count parameters were measured using an automated hematology analyzer (BC-6800, Mindray, Shenzhen, China). Serum C-reactive protein levels were measured using a Cobas c501 clinical chemistry analyzer (Roche Diagnostics, Basel, Switzerland). Both analyzers were routinely calibrated and used in accordance with the manufacturers’ instructions and the laboratory’s internal quality-control procedures.

### 2.4. Statistical Analysis

Data were entered into a database in IBM SPSS Statistics for Windows, Version 25.0 (IBM Corp., Armonk, NY, USA). The distribution characteristics of continuous variables were assessed using the Shapiro–Wilk test. Median and interquartile range (IQR) were used to summarize non-normally distributed data. Between-group differences were assessed using the Mann–Whitney U test. Categorical variables were summarized as frequencies and percentages and compared using the chi-square or Fisher’s exact test, as appropriate. Statistical significance was defined as a two-tailed *p* < 0.05. Binary logistic regression was performed to identify independent predictors of antibiotic administration, with CRP, gestational age, and weight at vaccination entered simultaneously as covariates. Model calibration was assessed using the Hosmer–Lemeshow goodness-of-fit test. Antibiotic administration was coded as 1 (treated) and 0 (not treated) as the dependent variable in the logistic regression model. Effect sizes (r) with 95% confidence intervals, derived from the Mann–Whitney U statistic via bootstrap resampling (1000 iterations), were calculated for the primary between-group comparisons.

## 3. Results

### 3.1. Demographic and Clinical Characteristics

**Figure 1:**
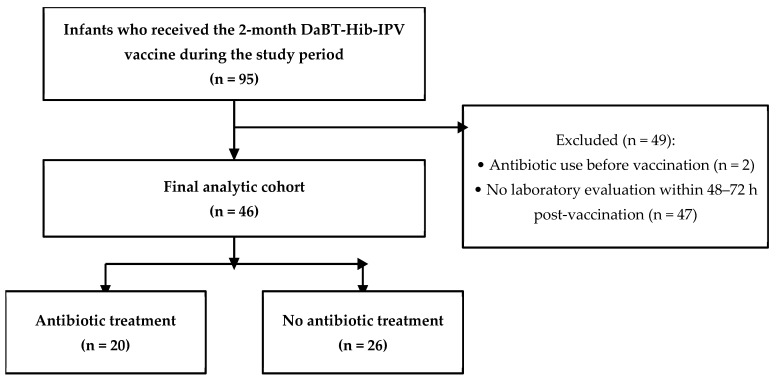
Study selection process.

During the study period, 95 infants received the 2-month DaBT-Hib-IPV vaccine while hospitalized in the NICU. Of these, 2 were excluded due to antibiotic use prior to vaccination and 47 due to the absence of laboratory evaluation within 48–72 h post-vaccination, yielding a final analytic cohort of 46 infants (Figure 1).

Of the 46 infants evaluated, 20 received antibiotic treatment after vaccination, while 26 were monitored without antibiotics. Sex distribution was similar between groups, and most infants were delivered by cesarean section. Medical charts also showed that both groups experienced similar frequencies of respiratory distress syndrome, surfactant use, and mechanical ventilation need. Hospital stays were significantly longer in the antibiotic group (median 99 vs. 78 days, *p* < 0.001) despite them having a significantly higher gestational age (31.5 vs. 28.0 weeks, *p* = 0.027). Infants in the antibiotic group trended towards having higher birth weight, although the difference was not significant (*p* = 0.075); however, the weight comparison reached significance when analyzed for measurements performed at the time of vaccination (median 2400 vs. 1880 g, *p* = 0.019) (Table 1).

### 3.2. Post-Vaccination Inflammatory Markers

**Figure 2:**
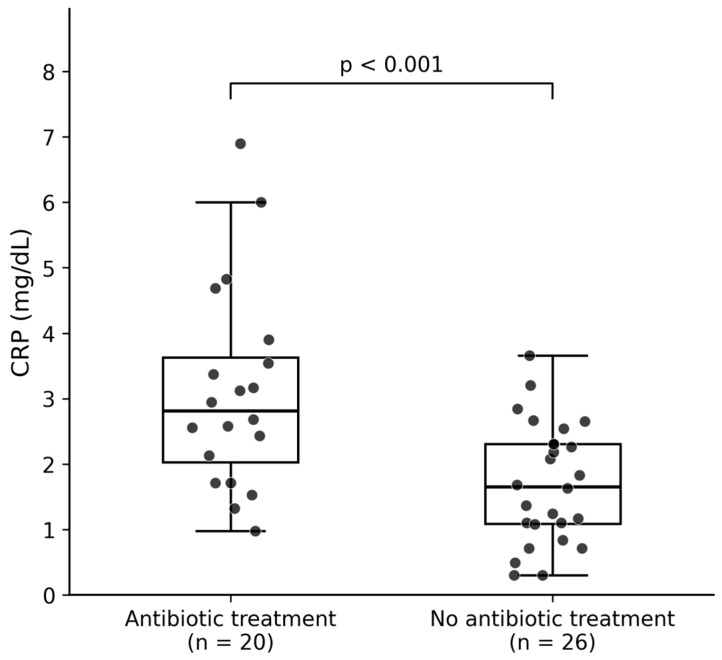
CRP levels after DaBT-Hib-IPV vaccination in antibiotic-treated versus untreated groups. Each point represents an individual infant; boxes indicate median and interquartile range.

Results from samples obtained after vaccination showed that WBC, platelet, neutrophil and lymphocyte counts as well as hemoglobin concentrations were similar for antibiotic recipients and non-recipients. The most pronounced difference was observed in CRP levels; with significantly higher CRP values in the antibiotic group compared with the non-antibiotic group (*p* < 0.001). Despite non-significant results for both underlying parameters, NLR was also found to be significantly elevated among recipients of antibiotic therapy (*p* = 0.041). However, SII values were similarly distributed across the groups (*p* = 0.666) (Table 2, Figure 2).

### 3.3. Culture Results and Antibiotic Treatments

Among the 20 recipients of antibiotic treatment after the DaBT-Hib-IPV vaccine, only three showed positive culture results (15.0%). *Staphylococcus epidermidis* was detected in two, and Klebsiella spp. was isolated in one case. No positive culture results were observed in the non-antibiotic group. In terms of antibiotics used among recipients, we found that vancomycin (65%) and amikacin (60%) were the most frequently administered antibiotics, followed by meropenem (40%) and piperacillin–tazobactam (35%). Empirical treatment was initiated using a dual-antibiotic regimen in all cases (Table 3). *Staphylococcus epidermidis* grew at 72 h of incubation in both positive cases; repeat blood cultures obtained during treatment were negative in both, suggesting a high likelihood of contamination rather than true bloodstream infection. However, per unit protocol, the full 10-day antibiotic course was completed irrespective of repeat culture result.

### 3.4. Predictors of Antibiotic Administration

CRP was the only independent predictor of antibiotic administration when the analysis was adjusted for gestational age and weight at vaccination (Exp(B) = 3.997, 95% CI 1.583–10.093, *p* = 0.003). Gestational age (*p* = 0.392) and weight at vaccination (*p* = 0.349) were not significant independent predictors. The model demonstrated acceptable fit (Nagelkerke R^2^ = 0.537; Hosmer–Lemeshow *p* = 0.302) (Table 4).

## 4. Discussion

Vaccines are designed to elicit a controlled, beneficial activation of the immune system, which is an expected and desirable physiological response rather than a pathological one. However, this same activation can transiently alter inflammatory markers, making them challenging to interpret in a population already at elevated risk of true nosocomial infection, especially so for patients already being monitored in the NICU. It is unclear whether classical inflammatory markers can be used to understand whether infection can be excluded in vaccine recipients. The DaBT-Hib-IPV vaccine is administered at 2 months and is therefore an early stimulus to the underdeveloped immune system. This study retrospectively evaluated 46 infants who received this vaccine and subsequently underwent clinical or laboratory assessment due to clinical deterioration or part of their routine monitoring. The present findings are consistent with two non-mutually exclusive possibilities: elevated inflammatory markers in some infants likely reflect an expected, transient immune response to vaccination, while in others they may reflect a true, evolving infection whose onset coincided temporally, but was not caused, by the vaccination. This study cannot distinguish between these possibilities on a per-patient basis, which is precisely the diagnostic ambiguity motivating this work. Our data suggest that CRP and the NLR were the only parameters that could at least partially discriminate between patients who were and were not administered antibiotics.

CRP is currently the most widely used first-line biomarker for the diagnosis of neonatal sepsis. However, CRP levels also rise in non-sepsis situations like necrotizing enterocolitis, intraventricular hemorrhage, hypoxia, shock, meconium aspiration, and surgery [20]. Furthermore, CRP measured early in the course of infection may not yet be elevated, potentially yielding false-negative results. In addition, CRP may react differently in early-onset and late-onset sepsis, and in premature and term newborns [9]. The median sensitivity and specificity of CRP for the detection of neonatal sepsis were respectively found to be 70% and 89% in a meta-analysis [21].

In the present study, despite significantly higher CRP levels in the antibiotic group, culture growth was confirmed in only 3 of 20 infants (15%), while the remaining 17 patients in this group received antibiotics either as a cautious approach or due to other supportive evidence based on clinical assessment. Nonetheless, we believe it is evident that CRP levels alone are insufficient in determining whether antibiotic initiation was necessary in some infants. This interpretation is supported by earlier work demonstrating that premature infants may develop abnormal CRP elevations after routine immunization, suggesting that post-vaccination CRP elevation may reflect vaccine-associated inflammation rather than sepsis [18]. With our logistic regression model, CRP was the only independent predictor of antibiotic administration when adjusted for gestational age and weight at vaccination, which could also indicate that the decision to initiate antibiotics were tied to CRP monitoring as well as clinical data. It should also be emphasized that antibiotic administration, rather than confirmed infection, was used as the dependent variable in the regression model; therefore, the model identifies factors associated with the clinical decision to treat rather than independent predictors of true infection.

Since no single biomarker reliably distinguishes vaccine-related inflammation from true sepsis [3], we examined the NLR and SII as composite measures. Regarding the SII, the present study found no significant difference between the two groups, which indicates that vaccine-related inflammation may alter the SII in a manner similar to true infection. On the other hand, NLR was indeed discriminatory in univariate analyses. This may be linked to neutrophil levels increasing in response to bacterial infection, and even though neutrophil counts alone were not discriminatory, the balance between neutrophils and lymphocytes appear to be impacted by the presence of true infection versus vaccine response [22,23]. As such, the significantly higher NLR values in the antibiotic group indicate an association between the NLR and the clinical decision to initiate antibiotic treatment; however, in the absence of a validated infection control group, this should not be interpreted as evidence that the NLR distinguishes true bacterial infection from vaccine-induced inflammation. A recent meta-analysis supports the NLR as a promising and readily available biomarker in neonatal sepsis, suggesting that the higher NLR observed in our antibiotic-treated group may indicate a relationship with clinical states that appear to require treatment [24]. Combined monitoring of markers may be crucial for accurate diagnosis of cases suspected to have neonatal infections, especially after administration of routine vaccinations. Indeed, it has been shown that monitoring procalcitonin, IL-6, CRP, and WBC could provide specific and sensitive evidence to identify infection in neonates [25], and a meta-analysis similarly reported that procalcitonin and CRP, but not WBC, distinguished neonatal sepsis effectively [26]. Furthermore, serum amyloid A appears to show notable diagnostic accuracy, in some cases superior to CRP or procalcitonin, in identifying neonatal sepsis and could be a useful tool in clinical practice [27,28,29]. Future studies may benefit from incorporating emerging biomarkers such as IL-6, IL-8, or serum amyloid A alongside conventional markers to improve diagnostic specificity in post-vaccination clinical deterioration [30].

In the NICU, unnecessary antibiotic exposure is increasingly recognized as a risk for both patient safety and antimicrobial resistance [31]. Consideration of antibiotic stewardship is crucial in all cases suspected to have bacterial infection; however, the tolerance against infection risks in neonates may be relatively low due to the critical nature of such cases. Nonetheless, clinicians must be aware that antibiotic resistance is an increasingly important contributor to neonatal mortality, particularly in low- and middle-income countries, where antibiotic-resistant neonatal sepsis carries a mortality rate approximately 30% higher than in high-income settings [32]. Antibiotics are the most frequently used medications in NICUs, and while antibiotic use has declined in some settings, the frequency and duration of treatment remain a concern for resistance development, especially in premature infants [33,34].

Clinical findings such as fever, irritability, or feeding difficulties after vaccination are insufficient to diagnose infection and must be interpreted alongside laboratory parameters. These findings are consistent with previously reported adverse events following combination vaccines [35,36]. DaBT-Hib-IPV frequently causes fever, irritability, poor appetite, and transient clinical deterioration. While crucial to monitor closely, these reactions are generally short-lived and tend to resolve spontaneously [36].

Importantly, these findings are applicable only to preterm infants remaining hospitalized in the NICU at the time of 2-month vaccination, and cannot be extrapolated to the general population of healthy, community-dwelling infants receiving routine immunization. This distinction is critical, as the immunologic and inflammatory baseline of a chronically hospitalized preterm infant differs substantially from that of a term infant vaccinated in an outpatient setting. The retrospective, single-center design of this study and the small sample size are the primary limitations of this study. The sample size limitation is linked to the fact that the target population was very specific—neonates who were monitored in the NICU and had received DaBT-Hib-IPV vaccination while being monitored. This study did not include pre-vaccination laboratory values, as routine blood testing is not performed in stable, growing preterm infants prior to the 2-month immunization in our unit. Consequently, the magnitude of the change induced by vaccination could not be determined, and a paired pre-/post-vaccination comparison was not possible. This represents an important limitation, and prospective studies incorporating baseline measurements are needed to clarify the true impact of vaccination on inflammatory markers. Furthermore, the study lacked a definitive reference standard for infection; culture positivity alone, particularly for coagulase-negative staphylococci, does not reliably distinguish contamination from true infection, and this should be considered when interpreting the association between biomarkers and antibiotic initiation. Consequently, this study cannot determine whether antibiotic-treated infants had a true, independently evolving infection that happened to fall within the post-vaccination observation window or whether their laboratory changes reflected vaccine-associated inflammation alone. The study design does not support, and should not be read as supporting, any causal relationship between vaccination and infection. The study population predominantly consisted of clinically stable, growing preterm infants; none required parenteral nutrition, invasive mechanical ventilation, or supplemental oxygen at the time of vaccination, and infants with active sepsis or recent major surgery were not represented. Clinical heterogeneity within the cohort was therefore limited. Nonetheless, due to the retrospective design, several potentially relevant variables were not systematically recorded and could not be incorporated into the adjusted analysis. It is crucial to note that despite these limitations, our study showed that CRP and NLR values showed an association with the antibiotic treatment decision. This association could indicate that these values align better with clinical findings triggering antibiotic treatment. However, the criteria guiding individual antibiotic decisions were not based on a standardized protocol due to the retrospective nature of the study, which introduces the possibility of selection bias.

## 5. Conclusions

Taken together, these findings demonstrate that higher CRP and NLRs were associated with antibiotic administration after vaccination, and prospective studies using standardized testing times and infection definitions are needed. Still, it is clear that close monitoring of these markers could improve clinical decision making. Utilizing other markers like the SII, procalcitonin, WBC, and NLR alongside CRP could contribute to reducing both antibiotic resistance and hospital stay duration by decreasing unnecessary antibiotic initiation. An increase in CRP or NLRs after vaccination may be observed as a potential risk factor for underlying infection and should prompt closer monitoring. However, these changes might be explained by a transient inflammatory response, making interpretations based on clinical findings, culture results, and additional biomarkers critical for management decisions

## Figures and Tables

**Table 1:** Comparison of demographic and clinical characteristics.

Parameter	Antibiotic Treatment		*p*-Value
	Yes (*n* = 20)	No (*n* = 26)	
Gestational age, weeks	31.5 (27.8–37.2)	28.0 (26.0–29.0)	**0.027 †**
Birth weight, g	1600 (1000–2680)	1130 (970–1220)	0.075
Male sex, *n* (%)	12 (60.0)	13 (50.0)	0.396
Cesarean delivery, *n* (%)	15 (75.0)	18 (69.2)	>0.999
APGAR score at 1 min	6.0 (6.0–7.0)	6.0 (5.0–6.0)	0.058
APGAR score at 5 min	8.0 (7.8–8.0)	8.0 (7.0–8.0)	0.595
Presence of RDS, *n* (%)	10 (50.0)	17 (65.4)	0.358
Surfactant use, *n* (%)	10 (50.0)	17 (65.4)	0.358
Mechanical ventilation, *n* (%)	19 (95.0)	24 (92.3)	>0.999
Presence of IVH, *n* (%)	4 (20.0)	7 (26.9)	0.786
Weight at vaccination, g	2400 (1958–3625)	1880 (1780–2080)	**0.019 †**
Length of hospital stay, days	99.0 (85.0–102.5)	78.0 (74.0–88.0)	**<0.001 †**

Data are presented as median (IQR). Mann–Whitney U test for continuous variables; chi-square or Fisher’s exact test for categorical variables. † *p* < 0.05. Abbreviations: APGAR, Appearance–Pulse–Grimace–Activity–Respiration; IVH, Intraventricular Hemorrhage; IQR, Interquartile Range; RDS, Respiratory Distress Syndrome. Mechanical ventilation and surfactant use refer to respiratory support received during the initial NICU admission, not at the time of vaccination; no infant required invasive mechanical ventilation, oxygen therapy, or parenteral nutrition at the time of vaccination.

**Table 2:** Comparison of laboratory data after vaccination.

Parameter	Antibiotic Treatment		*p*-Value
	Yes (*n* = 20)	No (*n* = 26)	
**White blood cell count (×10^3^/µL)**	9.33 (5.67–11.88)	9.50 (7.70–12.10)	0.283
**Hemoglobin (g/dL)**	9.10 (8.38–10.77)	8.90 (8.50–10.20)	0.560
**Platelet count (×1000/mm^3^)**	362.5 (170.8–413.8)	402 (337–491)	0.144
**Neutrophil count (×10^3^/µL)**	3.96 (3.10–4.39)	3.20 (1.86–5.16)	0.337
**Lymphocyte count (×10^3^/µL)**	3.80 (2.83–4.67)	4.20 (3.87–5.31)	0.098
**Neutrophil-to-lymphocyte ratio**	0.90 (0.74–1.53)	0.72 (0.40–0.97)	**0.041 †**
**CRP (mg/dL)**	2.82 (2.02–3.63)	1.63 (1.08–2.30)	**<0.001 †**
**SII**	300.85 (196.50–422.80)	264.30 (161.30–437.60)	0.666

Data are presented as median (IQR). The Mann–Whitney U test was used. † *p* < 0.05. Abbreviations: CRP, C-reactive protein; IQR, Interquartile Range; SII, Systemic Immune-Inflammation Index.

**Table 3:** Culture results and antibiotic therapies.

Parameter	*n* (%)
**Patients started on antibiotics**	20 (43.5)
**Patients with positive cultures**	3 (15.0)
**Microorganisms isolated**	
*Klebsiella* spp.	1 (33.3)
*Staphylococcus epidermidis*	2 (66.7)
**Antibiotics administered**	
Vancomycin	13 (65.0)
Amikacin	12 (60.0)
Meropenem	8 (40.0)
Piperacillin–tazobactam	7 (35.0)

All patients who received antibiotics were treated with dual combination therapy.

**Table 4:** Binary Logistic Regression Analysis for Predictors of Antibiotic Administration.

Variable	Beta	SE	Wald	*p*	Exp(B)	95% CI Lower	95% CI Upper
CRP (mg/dL)	1.386	0.473	8.594	**0.003**	3.997	1.583	10.093
Gestational age (weeks)	0.150	0.175	0.731	0.392	1.162	0.824	1.638
Weight at vaccination (g)	0.001	0.001	0.876	0.349	1.001	0.999	1.002
Constant	9.483	4.297	4.871	**0.027**	—	—	—

Nagelkerke R^2^ = 0.537; Hosmer–Lemeshow goodness-of-fit *p* = 0.302. Abbreviations: CI, confidence interval; CRP, C-reactive protein; Exp(B), odds ratio; SE, standard error. The effect size for the primary CRP comparison (Mann–Whitney U) was large (r = 0.490, 95% CI: 0.258–0.689).

## Data Availability

The data that support the findings of this study are available from the corresponding author upon reasonable request.

## Abbreviations

The following abbreviations are used in this manuscript:

APGAR	Appearance, Pulse, Grimace, Activity, Respiration
CI	Confidence Interval
CRP	C-Reactive Protein
DaBT-Hib-IPV	Diphtheria–Acellular Pertussis–Tetanus–Haemophilus influenzae Type B–Inactivated Poliovirus Vaccine
Exp(B)	Odds Ratio
IQR	Interquartile Range
IVH	Intraventricular Hemorrhage
NICU	Neonatal Intensive Care Unit
NLR	Neutrophil-to-Lymphocyte Ratio
RDS	Respiratory Distress Syndrome
SE	Standard Error
SII	Systemic Immune-Inflammation Index
WBC	White Blood Cell
